# MDMA-Assisted Therapy for Treatment-Resistant Posttraumatic Stress Disorder (PTSD) – One step further toward a patient-centered treatment pathway

**DOI:** 10.1192/j.eurpsy.2022.1731

**Published:** 2022-09-01

**Authors:** S. Pratas Penedos, M.J. Freire, I. Fonseca, A. Franco, N. Ribeiro, L. Moreno, M.M. Magalhães, P.A. Afonso, I.M. Alves, L. Paulino, C. Ramos, M.M. Figueiredo, L. Madruga, A. Gamito

**Affiliations:** Setúbal Hospital Center, Psychiatry And Mental Health, Setúbal, Portugal

**Keywords:** Psychotherapy, PTSD, MDMA (3,4-methylenedioxymethamphetamine), Trauma

## Abstract

**Introduction:**

PTSD is a chronic, debilitating condition with limited treatment efficacy. Accessing traumatic memories often leads to overwhelming distress, impacting treatment process. Current approved pharmacological treatments have exhibited small to moderate effects when compared with placebo. Evidence suggests 3,4,-methylene-dioxymethamphetamine(MDMA)-assisted psychotherapy as a viable option for refractory PTSD.

**Objectives:**

Comprehensive review of early clinical research, proposed mechanisms, safety and emerging therapeutic models.

**Methods:**

Eligible studies will be identified through strategic search of MEDLINE.

**Results:**

Pre-clinical and imaging studies suggest memory reconsolidation and fear extinction as candidate psychological and neurological mechanisms, involving MDMA’s combined effects of increasing serotonergic activity, as well the release of oxytocin and brain-derived neurotrophic factor in key memory and emotional circuits. Resulting reduction in amygdala and insula activation and increasing connectivity between the amygdala and hippocampus may create a “tolerance window” of neuroplasticity for emotional engagement and reprocessing of traumatic memories during psychotherapy. Early clinical trials report impressive and durable reduction in PTSD symptoms, with a safety profile comparable to that of SSRIs. A recently completed randomized, double-blind, placebo-controlled phase 3 trial reported full remission of PTSD symptoms in 67% of patients at 2 months, with no increase in suicidality, cardiovascular events or abuse behavior. Emerging treatment models underline the importance of unmedicated therapeutic sessions for preparation for the experience and subsequent integration as essential for full benefit and safety of the clinical context.

**Conclusions:**

The psychological impact associated with the COVID-19 pandemic is an reminder of the emotional and economic burden associated with PTSD. MDMA-assisted therapy may be a breakthrough approach meriting further multidisciplinary investment and clinical research.

**Disclosure:**

No significant relationships.

